# Increased prevalence of rotavirus among children associated gastroenteritis in Riyadh Saudi Arabia

**DOI:** 10.1186/1743-422X-8-548

**Published:** 2011-12-18

**Authors:** Hamsa T Tayeb, Hanan H Balkhy, Sameera M Aljuhani, Esam Elbanyan, Solaiman Alalola, Mohammad Alshaalan

**Affiliations:** 1 National Guard Health Affairs & King Abdullah International Medical Research Center, Research Genetic Laboratory, Riyadh, Saudi Arabia; 2 Infection Prevention and control Department, Riyadh, Saudi Arabia; 3 Division of Microbiology, Department of Pathology and Laboratory Medicine, Riyadh, Saudi Arabia; 4 Department of pediatrics, KAMC, Riyadh, Saudi Arabia

## Abstract

The aim of this study is to assess the epidemiology along with the molecular structure of rotavirus causing pediatric diarrhea among Saudi patients. However, in this report we sited the epidemiological reflect coming from our project.

**Methods:**

One thousand and seven diarrheal stool samples had been collected between Jan1st, 2008 and OCT 31st, 2010 from hospitalized patients below the age of 5 year. Samples were then examined using Enzyme-linked immunosorbent assay (ELISA). Demographic data were collected including age, sex, date of admission and discharge. Finally, the chi-squire test, α level of significance was used to test the variables in the data.

**Results:**

Of these 1007 stool samples, rotavirus was detected in 65.5% (660/1007 samples). We observed that children who are 1 year of age or less had more infection with rotavirus 81% (534/660) than those who is over 1 year of age (19%,126/660) (P = 0.000). Infections occur throughout the year with no clear significant seasonal peaks. The difference between males (57.5%, 380/660) and females (42.4%, 280/660) in terms of rotavirus positivity is statistically significant.

**Conclusions:**

The high rate of positivity, are at variance with previously published reports of rotavirus infection in Saudi Arabia since 2005 which reported a major decrease year by year in the incidence of rotavirus over; 2005, 2006 and 2008 with percentage of; 25%, 10%, 6% respectively explained by improvements in public health introduced in recent years. Our increasing rate result (65.5%) may suggest emerging of unusual serotypes, not been represent to our country earlier.

## Introduction

Rates of rotavirus illness among children in industrialized and less developed countries are almost similar, it is estimated that in developing countries severe dehydrating diarrhoea caused by HRV (Human rotavirus) results in an estimated 500,000 to 870,000 childhood deaths annually [1,2] and even in the developed world may account for over one million cases of diarrhoea each year [3-5]. However hard evidence of rotavirus-induced mortality is difficult to obtain.

Indicating that clean water supplies and good hygiene have little effect on virus transmission, so further improvements in water or hygiene are unlikely to prevent the disease. Thus the bulk of viral transmission must be presumably via person to person.

HRV reported in 14% (8135/58,110) to 42% (520/1,242) of cases of diarrhoea overall in KSA (Kingdom of Saudi Arabia) [6,7]. Recent studies further suggest that the incidence may be falling. Ghazi et al., (2005) who found that the incidence of rotavirus infections had decreased (10%) in the city of Makkah in 2005 [8]. Despite any apparent decrease in HRV incidence, Kheyami and his colleagues (2006) made a comparison study for the incidence in Saudi Arabia by reviewing of 22 studies published between 1982 to 2003 and concluded that HRV remains the most common cause of diarrhoeal infection in infant and young children in Saudi Arabia [9].

Most recently, Tayeb et al. addressed the molecular epidemiology of HRV, enteric adenovirus and astrovirus. In this study viruses were sought in faecal specimens and characterized for genotype using molecular methods for the first time in SA. Moreover, it includes the epidemiology of diarrhea viruses in the pediatric population over a period of one year in 2003 [10].

Therefore, further studies from Saudi Arabia, identifying the incidence of rotavirus and the peculiar environmental features of the country leading to a changing pattern of virus circulation, are needed.

## Materials and methods

### Stool collection

Label stool container with label, include patient unique identifier. Transfer at least one spoonful of stool into the labeled container and shut securely. Complete the specimen section in the case report form and feedback form. Send the specimen with the stool examination request and feedback form to the hospital lab.

### Preparation of stool extract

In order to analyze the samples, approximately 100 mg of each of the frozen specimen was thawed and diluted with 1 ml of Dulbecco's phosphate-buffered saline (PBS, pH 7.0) (ICN Biomedicals Inc., Ohio, USA), mixed gently in micro centrifuge tubes using minishaker, and clarified by centrifugation at 250 × g for 10 min at 4°C. One ml of the supernatant was recovered and divided into 250 μl aliquots, which were analyzed immediately or stored at −70°C until examined.

### Enzyme immunoassay for the detection of viruses antigens ELISA testing

One hundred μl of each extracted sample was tested for viral antigens using commercial ELISA kits (IDEA for rotaviruses) from DAKO (Cambridgeshire, UK) according to the manufacturer's instructions.

### Duration

Two years are the duration for the evaluation of seasonality of the infection among the patient population.

### Statistical analysis

The chi-squire test, α level of significance test were used when to analyze the season of infection, age distribution and the prevalence of rotavirus, infection. Moreover, Fisher's exact test (two-tailed) was used between Genders. P- Values less than 0.05 were considered significant

## Result

### Prevalence of rotavirus

The presence of rotavirus -positive stool samples was 65.5% (660/1007 samples) in (NGH) Riyadh.

ELISA-positive samples were used for further investigation which included molecular detection of rotavirus genotypes present in the ELISA positive samples.

An analysis for the age of children positive for rotavirus showed a significant increase in infection among the children who are 1 year of age or less with percentage of 81% (534/660) compared with those who are above 1 year of age (19%,126/660) (P = 0.000). Furthermore, the difference between males (57.5%, 380/660) and females (42.4%, 280/660) in terms of rotavirus positivity was statistical tendency, due to not be p < or = 0.05.

### Season of infection

**Figure 1:**
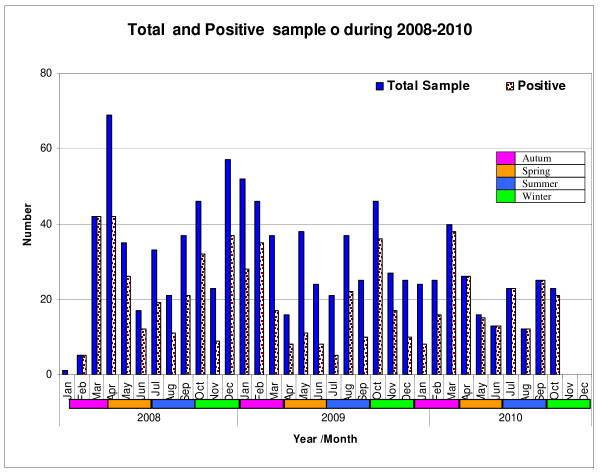
**Total and positive samples during 2008-2010**. Infections with rotavirus occur throughout the year with no significant seasonal peaks.

We analyzed seasonality of infection with these viruses. As can be seen from and Figure 1, Monthly distribution of positive samples during the year of collection for rotavirus (Group A). Infections occur throughout the year with no clear significant seasonal peaks.

## Discussion

**Figure 2:**
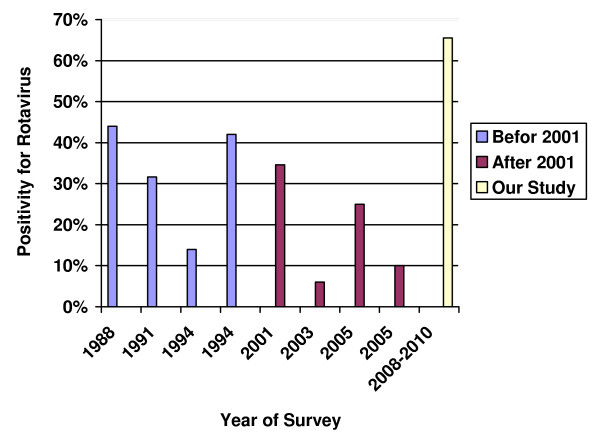
**Circulation of rotavirus within the country between 1988 to 2008**. RV reported in a high prevalence of diarrhoea cases overall in KSA during 1988 to 2001 [6,7,11,12]. Recent studies (after 2001) suggest that the incidence may be falling [8,10,13].The decrease in the incidence of rotavirus explained by improvements in public health introduced in recent years.

Since 2005 there were no data on the prevalence of HRV in our country. Thus, further study is needed in order to assess the extent of influence that each factor may have in the determination of virus introduction and circulation within the country and this would be best addressed by ongoing surveillance Figure 2.

However, our findings, 65.5% (660/1007) are at variance with previously published reports of rotavirus infection in Saudi Arabia since 2005. For example, Ghazi et al., 2005, Kheyami et al., 2006 and Tayeb et al., 2008 [8-10] reported a major decrease year by year in the incidence of rotavirus explained by improvements in public health introduced in recent years. Our explanation of this result may suggest emerging of unusual serotypes, not been represent to our country earlier, causing the increasing in the infection rate with Rotavirus. Therefore, our project aimed in its next phase especially at the detection of novel or unusual strains that may be emerging in the KSA such epidemiological data which will help to provide valuable insights into antigenic and genetic identities and possible sources of virus strains involved not only in individual acute pediatric gastroenteritis but also possible focal outbreaks in the community.

Many studies have shown the important role of rotavirus as a cause of diarrhoea in children in both developed and developing countries. Most of the cases occur in children less than 5 years of age, sometimes less than two [8,14] and in our case less than 1 year of age. We observed children infected with rotavirus who are 1 year of age or less 81% (534/660) be relatively more than those who are over 1 year of age (19%,126/660). Statistically significant with P-Value of = 0.000.

The city, from which samples were collected (Riyadh), represent the largest city and the political capital with colder, dry climate. It is the main location for government and subject to heavy traffic of people. Riyadh has a population of over 4.5 millions. Therefore, samples from this city represent subjects coming from different regions of the world. It signifies a desert climate and habit of nutritional style. It includes the major health institutions in the country with referral medical centers where patients are sent from different parts of the Kingdom.

Our data show a sustained incidence of rotavirus throughout the year, there is no obvious peak in winter and the peak in April does not coincide with either temperature minima or increases in rainfall. This is similar to findings elsewhere which reported in tropical and developing countries; diarrhoea occurs all year round, with a peak in summer. In Saudi Arabia, infection with rotavirus occurs the year around with no significant seasonal peak [10,13].

In conclusion incidence of rotaviruses detected appears to be higher than reported in the last few years and maybe continuing the upward trend in the incidence of this virus identified by others, if true, we hypothesis this to a new re assortment in the virus strain or to possible emerging of unusual strain in the kingdom. Analysis of rotavirus strains collected worldwide showed that the most common combination of rotavirus genotypes are G1[P8], G2[P4], G3[P8], and G4[P8] [15]. G1-G4 are the most common globally, accounting for almost all endemic rotavirus gastroenteritis [15,16]. Greater than 90% of children have developed antibody to group A rotavirus (G1-G4) by age 3 [17] and all have had at least one infection by age 5 [18]. Sequential acquisition of strains leads to increased immunity and the frequency of infection declines. Superimpose on this pattern are the epidemic strains, which typically include the more unusual viruses no history of exposure to these viruses and thus little protective immunity. Moreover, other G serotypes have now been found to be common in several other regions of the world, serotypes G5, G8 and G10 in Brazil [19], G9 in India [20] and, G12 in Brazil [21].

In order to elucidate this theory further investigation on the molecular characterization of rotavirus strain in the positive samples need to be conducted in the near future.

## Abbreviations

ELISA: Enzyme-Linked Immunosorbent Assay; KSA: Kingdom of Saudi Arabia; HRV: Human Rotavirus.

## Competing interests

The authors declare that they have no competing interests.

## Authors' contributions

HT is an Associate Scientist in KSAU-HS, KAIMRC-KAIMRC-R Department. Contributed in Data analysis, molecular testing, writing the paper. HHB is an Assistant Professor in KSAU-HS, KSAU-HS& KAIMRC Department. Contributed in Supervision of data entry. SAJ is an Assistant Professor in KSAU-HS, Microbiology-KAMC-R Department. Contributed in Supervision of data collection. EB is an Assistant Professor in KSAU-HS, Pediatrics-KAMC-R Department. Contributed in Supervision of data collection. SAA is an Assistant Professor in KSAU-HS, Pediatrics-KAMC-R Department. Contributed in Supervision of data collection. MA is an Assistant Professor in KSAU-HS, Pediatrics Department. Contributed in Supervision of data collection.
